# Association of Three Composite Inflammatory and Lipid Metabolism Indicators With Cardiovascular-Kidney-Metabolic Syndrome: A Cross-Sectional Study Based on NHANES 1999–2020

**DOI:** 10.1155/mi/6691516

**Published:** 2025-05-08

**Authors:** Jiayuan Song, Ziyi Xu, Han Yu, Aimin Li, Yiying Liu, Meiying Jin

**Affiliations:** ^1^College of Integrative Medicine, Changchun University of Chinese Medicine, Changchun, China; ^2^College of traditional Chinese Medicine, Changchun University of Chinese Medicine, Changchun, China; ^3^Department of Cardiology, Guang'anmen Hospital China Academy of Chinese Medical Sciences, Beijing, China

**Keywords:** cardiovascular-kidney-metabolic syndrome, CKM syndrome, lymphocyte to HDL-C ratio (LHR), monocyte to HDL-C ratio (MHR), neutrophil to HDL-C ratio (NHR)

## Abstract

**Background:** Various leukocyte-to-high-density lipoprotein cholesterol (HDL-C) ratios, namely the neutrophil to HDL-C ratio (NHR), lymphocyte to HDL-C ratio (LHR), and monocyte to HDL-C ratio (MHR), have been identified as potential inflammatory biomarkers. Despite this, the intricate relationship between these ratios and Cardiovascular-Kidney-Metabolic (CKM) Syndrome has yet to be fully elucidated. This study aims to explore the associations between these white blood cell ratios and the presence of CKM Syndrome.

**Methods:** This cross-sectional retrospective analysis utilized data from 19,534 individuals diagnosed with CKM Syndrome, sourced from the National Health and Nutrition Examination Survey (NHANES) database covering the years 1999–2020. Participants were stratified, and relevant covariates were adjusted during the analysis. Weighted logistic regression models were employed to statistically assess the relationships between the inflammatory markers and the differing stages of CKM Syndrome, with stage 0 serving as the reference point.

**Results:** After adjusting for all the covariates, high levels of three inflammatory indicators were associated with higher odds of having CKM Syndrome stage 1–4, using stage 0 as a reference. When we assessed the associations between inflammatory indicators with stage 3–4 with stage 0–1–2 as the reference group, we found that inflammatory indicators still increased the risk of higher CKM Syndrome stage. The dose–response relationship revealed that the inflammatory indicators increased the risk of higher CKM Syndrome stage. After conducting subgroup analyses, we found that LHR and education, as well as LHR, MHR, and drinking status, had significant interactions.

**Conclusion:** Elevated NHR, LHR, and MHR are significantly associated with an increased risk of CKM Syndrome across stages 1–4.

## 1. Introduction

Cardiovascular-Kidney-Metabolic (CKM) Syndrome is a complex clinical syndrome that affects the heart, kidneys, and metabolic system, and its harmfulness is undeniable [1]. Inflammation, oxidative stress, insulin resistance, and vascular dysfunction are key contributors to metabolic risk factors, kidney disease progression, exacerbation of cardio-renal interactions, and the development of cardiovascular disease (CVD) [2]. In the last 5 years, both the incidence and mortality rates of CKM Syndrome have risen steadily, presenting a major challenge to global public health. Large-scale cohort studies show that the risk of all-cause mortality for stages 1–4 of CKM Syndrome is 1.24, 1.72, 2.58, and 3.73 times higher than for stage 0, respectively [3]. These findings highlight the significant threat CKM Syndrome poses to human health, especially considering the complex interactions among metabolic abnormalities, chronic kidney disease (CKD), and CVDs [4]. At present, clinical practice has not yet achieved early detection and treatment of CKM Syndrome. Therefore, identifying a biomarker associated with the pathogenesis of CKM Syndrome is crucial for its early detection.

Recent studies suggest that the neutrophil-to-HDL-C ratio (NHR), lymphocyte-to-HDL-C ratio (LHR), and monocyte-to-HDL-C ratio (MHR) could be biomarkers for inflammation and lipid metabolism [5–10]. These ratios allow for a detailed analysis of their individual effects and improve our understanding of their interactions, uncovering the complex mechanisms of these physiological processes. Previous research has validated the predictive value of these ratios in cardiometabolic diseases and nephropathy, including hypertension, cardiovascular risk, and acute kidney injury [11–16]. These findings highlight the sensitivity of the biomarkers but also raise questions about their specificity for diagnosing different stages of CKM Syndrome in the US population.

No studies to date have thoroughly investigated the relationship between the ratios of NHR, LHR, and MHR and CKM Syndrome. This study aims to assess the potential of NHR, LHR, and MHR as biomarkers for CKM Syndrome. We tested the hypothesis that higher levels of these ratios correlate with a higher likelihood of developing various stages of CKM Syndrome. The primary objective was to employ stringent statistical methods to control for potential confounders, utilizing cross-sectional NHANES data from 1999 to 2020, to determine if biomarkers are linked to the risk of CKM Syndrome and its stages.

## 2. Methods

### 2.1. Study Design and Population

**Figure 1:**
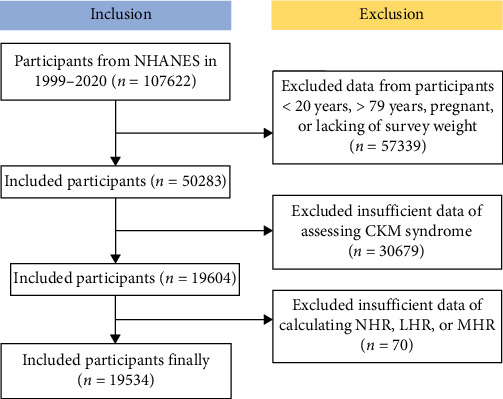
Screening flow of respondents.

The National Health and Nutrition Examination Survey (NHANES) is a national survey that collects comprehensive data on nutrition and health across the United States population, conducted biennially via a cross-sectional study design employing a sophisticated multistage probability sampling technique [17]. This study employs the NHANES dataset, which is publicly available and has been collected following ethical guidelines, including securing informed consent from all participants. The experimental designs and associated NHANES data are available on a publicly accessible platform: www.cdc.gov/nchs/nhanes/. Each methodological approach adhered strictly to the pertinent ethical standards and regulations. This study analyzed the Laboratory data set of the NHANES from 1999 to 2020 in the United States, initially including 107,622 participants [18]. Exclusions were made for 57,339 participants who were aged <20 or >79 years, pregnant, or lacking survey weight, and for 30,679 participants with insufficient data of assessing CKM Syndrome, and for 70 participants with missing data for NHR, LHR, and MHR. Ultimately, 19,534 participants were included in the study (Figure 1).

### 2.2. Diagnosis of CKM Syndrome Stage 0–4

The American Heart Association (AHA) advises dividing CKM Syndrome into five stages based on pathophysiological mechanisms, disease risks, and opportunities for prevention [1]. The diagnostic criteria we refer to are based on the standards from the AHA and have been adjusted according to NHANES [19]. More detailed content was displayed in Tables S1 and S2.

Stage 0: Lack of risk factors for CKM Syndrome, emphasizing primary prevention and maintaining cardiovascular health.

Stage 1: Identified by an excess of or malfunctioning adipose tissue, including abdominal fat and overweight/obesity.

Stage 2: CKD and metabolic risk factors, such as metabolic syndrome, hypertension, and moderate-to-high-risk CKD, are present.

Stage 3: CKM Syndrome is linked to preclinical cardiovascular illness, such as asymptomatic heart failure and subclinical atherosclerotic CVD.

Stage 4: CKM Syndrome is linked to clinical CVD, such as peripheral arterial disease, heart failure, stroke, and coronary artery disease (CAD).

Although there are differences in disease severity, both stages 3 and 4 represent the advanced stages of CKM Syndrome and share similar pathophysiological mechanisms. We defined stages 0–2 as nonadvanced CKM Syndrome, and stages 3–4 as advanced CKM Syndrome, and considered CKM stage as a binary variable [20]. Notably, we applied the CKD-EPI formula from 2021 [21]. More detailed content was displayed in Table S3.

### 2.3. Definition of NHR, LHR, and MHR

These ratios constitute a new class of inflammatory biomarkers indicative of systemic inflammation and lipid metabolism, and they have been associated with the prognostic outcomes of various diseases. High-density lipoprotein cholesterol (HDL-C) concentrations were measured utilizing the Roche Cobas 6000 and Roche modular P chemical analyzers. The calculations for these inflammatory markers are articulated as follows:

NHR: Calculated as the ratio of neutrophil count to HDL-C.

LHR: Calculated as the ratio of lymphocyte count to HDL-C.

MHR: Calculated as the ratio of monocyte count to HDL-C.

### 2.4. Demographic Characteristics and Other Covariates

Covariates across three dimensions; sociodemographic, life behavior variables, and chronic diseases, were identified as potential innate confounding factors. Sociodemographic characteristics included gender (female or male), age groups (20–39, 40–59, and ≥60 years), race/ethnicity (Mexican American, other Hispanic, non-Hispanic white, non-Hispanic black, and other race, including multiracial), and the levels of educational attainment (less than high school, high school graduate/GED or equivalent, and higher than high school). The poverty income ratio (PIR) serves as a measure of income in relation to the federal poverty threshold, taking into account factors such as economic inflation and family size. Marital status was divided into three distinct categories: those who are married or cohabiting with a partner; individuals who are widowed, divorced, or separated; and those who have never married. Life behavior variables contained smoking status, drinking status, and physical activity. Smoking status was categorized into two groups: current nonsmokers and current smokers. Alcohol consumption was classified into three distinct levels: current heavy drinkers, current moderate drinkers, and current nondrinkers. Physical activity was considered as walking, cycling, exercising, and leisure pursuits. Chronic diseases were excluded from the covariate analysis due to the focus of this study on CKM Syndrome, which integrates cardiac, renal, and metabolic diseases.

### 2.5. Statistical Analysis

Considering the multistage complex sampling in the NHANES database, we applied a mobile examination center (MEC) exam weight for all analyses [22]. We compared the differences in variable distribution across five CKM Syndrome stages. Continuous variables were expressed as weighted mean ± standard deviation (SD) and analyzed by the weighted Analysis of Variance (ANOVA). Categorical variables were expressed as numbers (weighted proportions) and analyzed applying weighted chi-square test [23]. Missing values of covariates were treated by multiple imputation in the “MICE” package. Due to NHR, LHR, and MHR following a skewed distribution, we Log2-transformed them in our study. CKM Syndrome stage was recognized as an ordered categorical variable, with higher levels indicating more severe illness. Therefore, an ordinal logistic regression model is suitable for analysis in the CKM Syndrome stage. However, the data did not pass the proportional odds assumption test, and we assessed the associations between NHR, LHR, and MHR and four CKM Syndrome stages with stage 0 as the reference group in a weighted logistic regression model [1]. NHR, LHR, and MHR were used in continuous analyses, and then they were divided into quartiles (Q1–Q4) for categorical analyses. We also assessed the associations of NHR, LHR, and MHR with stage 3 and 4 with stage 0, 1, and 2 as the reference group. To evaluate the potential nonlinear correlation between three inflammatory indicators and CKM Syndrome stages, we established restricted cubic spline (RCS) regression models with three knots at the 10^th^, 50^th^, and 90^th^ percentiles [24]. Subgroup analyses were employed to evaluate whether there is an interaction between inflammatory markers and grouping variables on the progression of CKM Syndrome. Likelihood ratio tests were used to assess interactions [25]. All the models were adjusted for the covariates as mentioned earlier and conducted in R (version 4.3.0). Two-tailed *p* values < 0.05 were considered statistically significant.

## 3. Results

### 3.1. Characteristics of Study Objects

The basic characteristics of 19,534 participants from NHANES 1999–2020 were revealed in Table 1. Approximately half (51.2%) were male, and the largest proportion of participants were aged 40–59 (41%). In addition, the number of participants in stage 2 is the highest. There were significant differences between the five groups in terms of all variables (Table 1).

### 3.2. The Associations Between NHR, LHR, and MHR and CKM Syndrome Stages in Weighted Logistic Regression Model

As displayed in Table 2, after adjusting for all the covariates, a high level of three inflammatory indicators was associated with higher odds of having CKM Syndrome stages 1, 2, 3, and 4, either in continuous analyses or categorical analyses (*p* and *p* trend < 0.001). When we assessed the associations between inflammatory indicators with advanced stages, we found that inflammatory indicators still increased the risk of higher CKM stages (*p* and *p* trend < 0.001).

### 3.3. The Associations Between NHR, LHR, and MHR and CKM Syndrome Stages in RCS Regression Model

**Figure 2:**
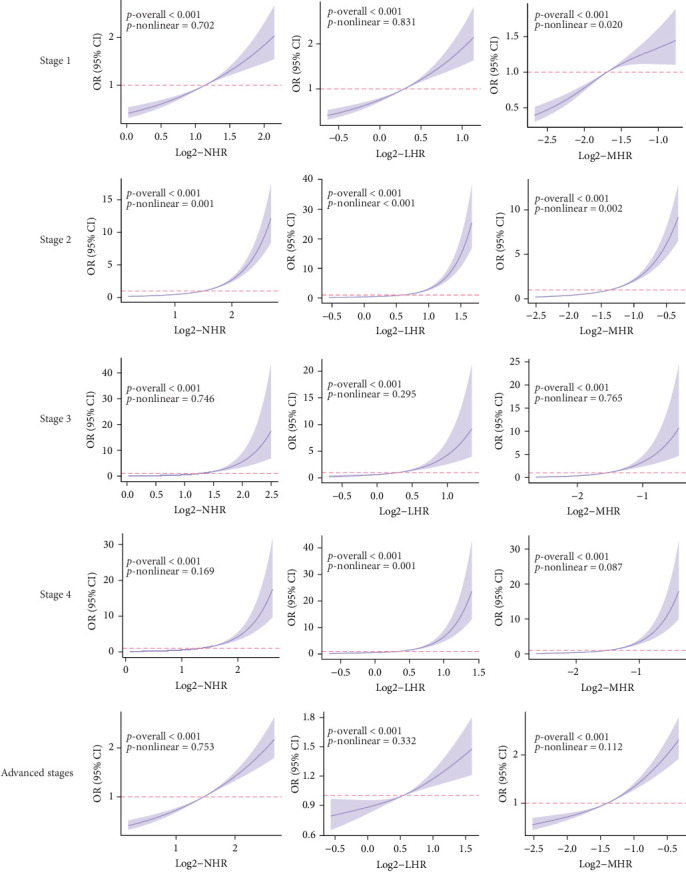
The RCS regression model for the three indicators across different stages of CKM Syndrome.

The dose–response relationship revealed that the inflammatory indicators increased the risk of higher CKM Syndrome stage (Figure 2). Specifically, the correlations between NHR and stage 1, 3, 4, and advanced stages, LHR and stages 1,3, and advanced stages, and MHR and stages 3, 4, and advanced stages are linear (all nonlinear *p* > 0.05). The correlations between NHR and stage 2, LHR and stage 2 and 4, and MHR and stage 1 and 2 are nonlinear (all nonlinear *p* < 0.05).

### 3.4. Subgroup Analyses

**Figure 3:**
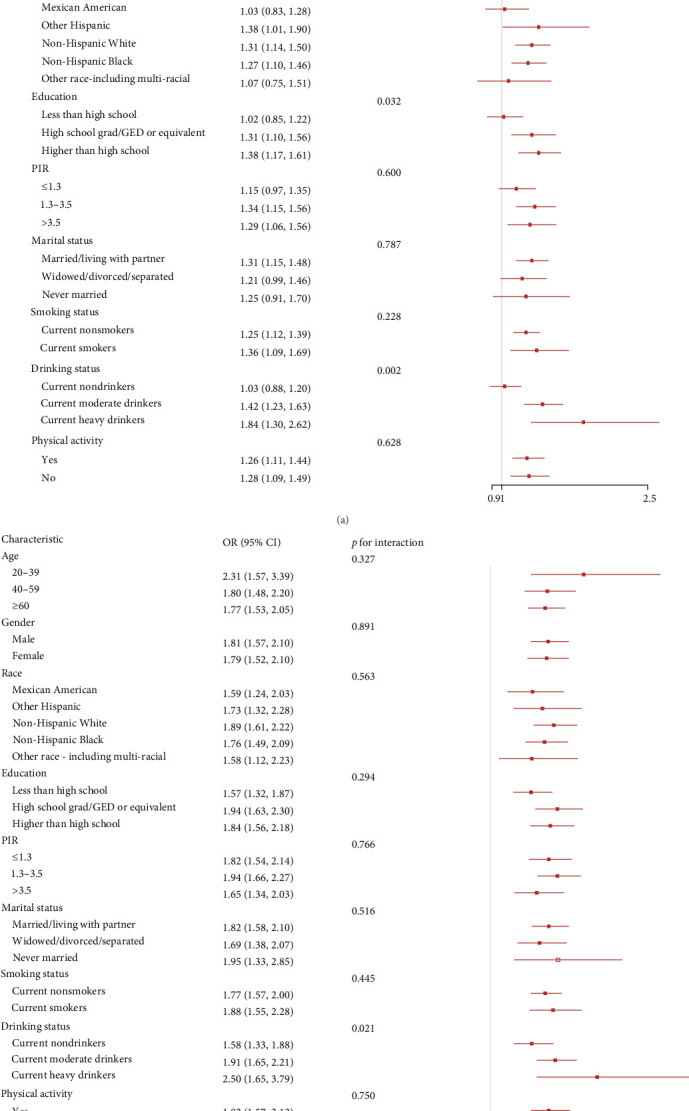
The models in the subgroup analyses were adjusted for gender, age, race, education, poverty-to-income ratio (PIR), marital status, drinking status, smoking status, and physical activity. (A) Associations between LHR and advanced CKM stages. (B) Associations between MHR and advanced CKM stages.

We explored the associations of inflammatory indicators with advanced CKM stages according to different groups of gender, age, race, education, PIR, marital status, drinking status, smoking status, and physical activity (Figure 3 and Figure S1). In the vast majority of groups, three inflammatory indicators were positively with advanced CKM stages, consistent with the analysis results above. We found that LHR and education had a significant interaction. In addition, LHR, MHR, and drinking status also had a significant interaction (*p* for interaction < 0.05).

### 3.5. Discussion

This study represents the first comprehensive examination of the relationship between combined inflammatory and lipid metabolism markers and CKM Syndrome. The ratios of NHR, LHR, and MHR function as integrative indicators of systemic inflammation, merging HDL levels with results from complete blood count tests. This methodology affords a holistic perspective on inflammation and lipid metabolism, which are critical elements in the pathophysiology of CKM Syndrome [26]. We performed a comprehensive statistical analysis on data from 19,534 participants in the NHANES study, revealing a statistically significant correlation between high levels of NHR, LHR, and MHR and the risk of developing CKM Syndrome.

It should be emphasized that the specific biological pathogenic mechanisms of CKM Syndrome, a complex disease affecting the cardiovascular, renal, and metabolic systems, remain unclear. Based on previous studies, we hypothesize that inflammatory responses, insulin resistance, and dysregulated lipid metabolism are potential mechanisms. Inflammation is not only a hallmark of CKM Syndrome but also plays a key role in its development. CRP and IL-6 contribute to both atherosclerosis and kidney damage [27]. This inflammation activates JAK/STAT and MAPK pathways, affecting vascular smooth muscle cell behavior and promoting CVD [28]. Oxidative stress can also trigger inflammatory responses in the body, with ROS overproduction causing cellular and DNA damage, correlating with the progression of cardiovascular and renal diseases [27]. Insulin resistance is another key factor in CKM Syndrome, associated with the disruption of insulin signaling by lipids and inflammation. Insulin resistance increases the risk of type 2 diabetes and is linked to hypertension and CVDs [29]. Insulin resistance also promotes metabolic dysregulation, which accelerates the start and progression of CKM Syndrome by causing endothelial dysfunction, vascular inflammation, and atherosclerosis. Lipid metabolism dysregulation leads to increased triglyceride levels and decreased HDL-C, both of which are key risk factors for atherosclerosis. Lipotoxicity, caused by the toxic effects of excess lipids on cells, leads to endothelial dysfunction, inflammation, and vascular damage [4, 30]. Endothelial dysfunction, an early sign of CKM Syndrome, relates to lipid metabolism dysregulation and inflammation. It hinders vascular relaxation, promoting thrombosis and atherosclerosis [31].

Among these, HDL is not only a key mediator of reverse cholesterol transport (RCT) but also plays a central role in the cross-regulation of lipid metabolism and inflammation. HDL is a heterogeneous group of particles with diverse lipid and protein compositions, including cholesterol, phospholipids, triglycerides, and proteins such as apolipoprotein A-I and A-II [32]. HDL-C measured in clinical practice reflects the combined concentration of cholesterol and cholesterol esters in HDL, but not the full range of its lipid components. However, HDL particles can contain hundreds of different lipids that are not measured in standard clinical HDL cholesterol tests [33]. We believe that the function of HDL extends far beyond its cholesterol content (HDL-C), as it acts as a carrier for proteins, miRNAs, and metabolites, profoundly influencing the pathophysiological processes of the heart and kidneys. HDL-associated apolipoproteins (e.g., ApoA-I and ApoM) and enzymes (e.g., paraoxonase 1 and PON1) can inhibit oxidative stress and the release of proinflammatory cytokines. In CKD patients, the decreased antioxidant capacity of HDL is associated with an increased risk of cardiovascular events [34, 35]. HDL can bind and transport various miRNAs (e.g., miR-223 and miR-126), which regulate the functions of target cells (e.g., endothelial cells and glomerular mesangial cells) through paracrine or endocrine mechanisms. miR-223 can inhibit the expression of ICAM-1 in endothelial cells, reducing the formation of atherosclerotic plaques [36, 37]. miR-126 inhibits inflammation and regulates angiogenesis. However, in CKD patients, decreased HDL-miR-126 levels may lead to enhanced inflammatory responses, abnormal angiogenesis, and more severe renal vascular damage, thereby promoting CKD progression [38]. HDL metabolism is regulated by various factors, including the activities of lipases such as hepatic lipase (HL) and endothelial lipase (EL). HL and EL hydrolyze triglycerides and phospholipids in HDL, causing HDL particles to shrink and ApoA-I to be released and cleared by the kidneys [39]. Moreover, HDL metabolism is closely related to energy metabolism, as its lipid components can serve as energy sources and participate in intracellular metabolic processes [40]. HDL is also associated with the metabolism of various bioactive molecules, such as sphingosine-1-phosphate (S1P) and oxidized phospholipids (ox-PL), which play important roles in cell signaling and inflammatory responses [41, 42].

In recent times, numerous clinical studies have demonstrated connections between composite markers of inflammation and lipid metabolism, as well as the onset and progression of cardiovascular, renal, metabolic diseases, and obesity. Obesity is one of the metabolic disorders identified. Marra noted that individuals with metabolic syndrome (MetS+) had significantly higher levels of MHR, LHR, NHR, platelet/HDL-C ratio (PHR), and system inflammation response index (SIRI) compared to those without MetS. These increased levels showed a positive correlation with the severity of MetS [43]. Kohsari emphasized the notable relationships between MHR and LHR concerning MetS and diabetes. Moreover, both MHR and LHR exhibited a significant positive link with cardiometabolic risk factors. In women, inflammatory biomarkers are closely related to cardiometabolic risk factors [15]. Pan identified a significant relationship between elevated NHR and a higher risk of CVD, particularly among males, in contrast to those with low NHR and females. Correlation studies indicated that NHR positively related to several anatomical and functional measures, such as aorta, left atrium, right atrium, right ventricle, end systolic diameter of left ventricle, end diastolic diameter of left ventricle, main pulmonary artery, right ventricular outflow tract, interventricular septum, and left ventricular posterior wall, while showing an inverse relationship with the E/A ratio, thus indicating a connection to cardiovascular risk [12]. Chuang pointed out that both NHR and NLR are useful for pinpointing individuals at heightened risk for CVD. When evaluating their combined effects, NHR alone demonstrates greater predictive capability for CVD prognosis than NLR or the combination of both markers [44]. Furthermore, Wu discovered that elevated levels of urinary tungsten are associated with an increased risk of CVD. MHR, in conjunction with MC, WBC, and HDL, plays a mediating role in the relationship between urinary tungsten and CVD, with MHR having the most pronounced effect. This indicates that MHR should be prioritized for future intervention strategies [45]. However, these indicators have not been confirmed to be associated with CKM Syndrome. Our research primarily investigates the following three aspects.

First, after adjusting for covariates, our research demonstrates that individuals in stages 1–2 of CKM Syndrome have lower levels of NHR, LHR, and MHR compared to stage 0. The risk associated with CKM Syndrome appears to escalate in conjunction with increasing levels of NHR, LHR, and MHR, thereby indicating a significant relationship between these markers and the heightened risk of CKM Syndrome. This finding was validated in both continuous and categorical analyses, indicating that these composite inflammatory and lipid metabolism markers are intimately linked to the progression of CKM Syndrome. These results underscore the significance of inflammation and lipid metabolism in the development of CKM Syndrome and offer potential biomarkers for early detection and intervention.

Second, CKM Syndrome is a complex clinical condition that affects the heart, kidneys, and metabolic system. The severity and manifestations of CKM Syndrome vary significantly across its different stages. A key distinction between the advanced stages and the early stages 0–2 is the extent of cardiac and renal involvement. Patients in the early stages typically do not exhibit signs of heart disease, whereas those in the advanced stages often present with subclinical and clinical CVDs. This discrepancy may be associated with changes in inflammatory and lipid metabolism indicators. Research has established that heightened inflammatory markers correlate with an elevated risk of CKM Syndrome [46]. Moreover, disorders in lipid metabolism significantly contribute to the progression of CKM Syndrome, particularly in its later stages [27, 28, 47]. Further analysis reveals that inflammatory markers are correlated with an increased risk in the advanced stages 3–4, even when the early stages 0–2 are used as a reference. The increasing levels of NHR, LHR, and MHR as CKM Syndrome progresses indicate that these factors could intensify damage to both the heart and kidneys, thus worsening the syndrome's severity. Liu found that MHR and NHR possess similar capabilities in forecasting the presence and degree of CAD. Among patients experiencing chest pain, elevated levels of MHR and NHR combined with reduced HDL-C levels were recognized as risk factors for significant stenosis, in contrast to LHR [48]. These elements were similarly linked to the severity of coronary stenosis in individuals with both anxiety disorders and chest pain [49]. Lin revealed that an increase in MHR alongside BMI is associated with an elevated risk of cardiorenal syndrome (CRS). Both high MHR and obesity act as standalone risk factors for CRS, with a greater occurrence noted in those displaying both conditions [50]. This aligns with our research outcomes. This evidence suggests that within the context of CKM Syndrome, levels of NHR, LHR, and MHR are significantly elevated in patients suffering cardiac and renal dysfunction, providing innovative research perspectives into CKM Syndrome's progression. Early identification and intervention based on these markers could be crucial in mitigating the impact of CKM Syndrome on cardiac and renal health.

Third, after conducting subgroup analyses, we identified a significant interaction between LHR, MHR, and alcohol consumption status. The analyses indicated that this interaction may be associated with the chronic effects of alcohol on lipid metabolism and inflammation. Research has shown that the consumption of alcohol can lead to reduced serum levels of HDL-C and apolipoprotein A–I, which may, in turn, worsen atherosclerosis [51]. Additionally, the ways in which alcohol is consumed exhibit correlations with the likelihood of developing metabolic syndrome and CVDs. High levels of alcohol intake could be associated with an increased risk of both metabolic syndrome and cardiovascular conditions, while low levels of consumption seem to correlate with a decreased risk [52, 53]. This suggests that alcohol might affect the likelihood of CKM Syndrome through various mechanisms. Mostofaky identified a dose–response relationship linking alcohol intake to CVD risk, noting that heavy drinking was related to an increased risk of cardiovascular issues the following day (~6–9 drinks: RR (95% CI) = 1.3–2.3) and the subsequent week (~19–30 drinks: RR (95% CI) = 2.25–6.2) [54]. These findings underscore the significance of taking lifestyle factors into account when evaluating the risk of CKM Syndrome and could aid in the development of targeted prevention and intervention strategies for specific populations. For example, in individuals with a higher predisposition to CKM Syndrome, if they are identified with elevated LHR, MHR, and a habit of consuming alcohol, particularly in large quantities, it can be recommended that they reduce their alcohol consumption as one of the preventive or interventive measures for the syndrome. Concurrently, promoting healthy alcohol consumption habits (such as moderate drinking or abstinence) among the general population may also contribute to lowering the risk of developing CKM Syndrome.

### 3.6. Strengths and Limitations

This study has several strengths. Firstly, it explores the association between NHR, LHR, MHR, and CKM for the first time, providing clues for the prevention and treatment of CKM. Secondly, it conducted a weighted analysis based on the complex sampling design of NHANES. Utilizing the sampling weights, stratification variables, and clustering variables provided by NHANES ensured that the study was representative of the national population and corrected for sampling bias and nonresponse issues. Thirdly, the use of complex statistical models such as weighted logistic regression and RCS enabled a detailed exploration of the linear and nonlinear associations between biomarkers and stages of CKM Syndrome. Thanks to this rigorous methodology, the observed associations are less susceptible to interference from unmeasured or residual variables.

However, the study does present certain limitations that merit consideration. Primarily, due to its cross-sectional design, which captures a single-instance snapshot rather than a longitudinal view, causality cannot be established. This highlights the necessity for prospective studies to further investigate these findings. Additionally, as the analysis relies solely on the NHANES dataset, the trends observed predominantly reflect the U.S. population, which may limit the generalizability of results to diverse global populations. Furthermore, by focusing exclusively on three indicators related to lipid metabolism and inflammation, the study may overlook other potentially predictive biomarkers. There is also the possibility that unmeasured factors may influence the results, despite controlling for known confounders. Future research should delve deeper into the complex relationships between CKM Syndrome and associated mortality.

## 4. Conclusions

In summary, our study demonstrates a positive correlation between NHR, LHR, MHR, and the rising incidence of CKM Syndrome, identifying these markers as potential novel predictors. This lays a foundation for early CKM Syndrome prevention in at-risk groups. Clinicians can use a panel of inflammatory and lipid metabolism markers to detect at-risk individuals and improve screening efficiency. Moreover, future studies should validate our findings with rigorous, high-quality research.

## Figures and Tables

**Table 1:** Characteristics of Participants grouped by CKM stages in NHANES 1999–2020.

Variables	Total	Stage					*p*
		Stage 0	Stage 1	Stage 2	Stage 3	Stage 4	
*n*	19,534	1806	3804	10,973	1259	1692	—
Age, *n* (%)	—	—	—	—	—	—	<0.001
20–39	6,340 (35.2)	1,230 (64.6)	1830 (47)	3,195 (30.9)	12 (1.1)	73 (5.2)	—
40–59	7,141 (41)	477 (30.5)	1,432 (39.7)	4,698 (47.5)	83 (7.5)	451 (30.2)	—
≥60	6,053 (23.8)	99 (4.9)	542 (13.3)	3,080 (21.6)	1,164 (91.4)	1,168 (64.6)	—
Gender, *n* (%)	—	—	—	—	—	—	<0.001
Male	10,027 (51.2)	713 (37.2)	1905 (51.4)	5,633 (52.6)	771 (58.4)	1,005 (58.7)	—
Female	9,507 (48.8)	1,093 (62.8)	1899 (48.6)	5,340 (47.4)	488 (41.6)	687 (41.3)	—
Race, *n* (%)	—	—	—	—	—	—	<0.001
Mexican American	3,445 (8.1)	225 (5.7)	734 (10.3)	2,073 (8.5)	215 (6.1)	198 (4)	—
Other Hispanic	1744 (5.6)	133 (4.7)	387 (6.8)	991 (5.6)	101 (5.3)	132 (3.9)	—
Non-Hispanic White	8,304 (68.8)	939 (74.6)	1,463 (65.1)	4,552 (68.6)	526 (68.2)	824 (72.4)	—
Non-Hispanic Black	4,121 (10.4)	285 (7.6)	772 (10.7)	2,321 (10.5)	324 (13.8)	419 (11.9)	—
Other race, including multiracial	1920 (6.9)	224 (7.4)	448 (7.1)	1,036 (6.7)	93 (6.6)	119 (7.8)	—
Education, *n* (%)	—	—	—	—	—	—	<0.001
Less than high school	4,997 (16.2)	276 (10.4)	792 (13.2)	2,882 (16.9)	465 (25)	582 (24.7)	—
High school grad/GED or equivalent	4,430 (23.7)	334 (18)	781 (21.4)	2,608 (25.1)	314 (28)	393 (26.2)	—
Higher than high school	10,107 (60)	1,196 (71.6)	2,231 (65.4)	5,483 (58)	480 (47)	717 (49.1)	—
PIR, *n* (%)	—	—	—	—	—	—	<0.001
≤1.3	5,775 (20.3)	445 (17.2)	1,013 (18)	3,277 (20.7)	400 (23.3)	640 (27.2)	—
1.3–3.5	7,591 (36.4)	651 (32.1)	1,479 (36.6)	4,227 (36.2)	585 (45.8)	649 (39.6)	—
>3.5	6,168 (43.3)	710 (50.7)	1,312 (45.4)	3,469 (43.1)	274 (30.9)	403 (33.2)	—
Marital status, *n* (%)	—	—	—	—	—	—	<0.001
Married/living with partner	12,178 (66)	990 (58.4)	2,392 (66.5)	6,995 (67.2)	762 (62.8)	1,039 (68.8)	—
Widowed/divorced/separated	3,892 (17)	173 (9.8)	544 (13)	2,220 (17.7)	434 (32.7)	521 (25.4)	—
Never married	3,464 (17)	643 (31.8)	868 (20.5)	1758 (15)	63 (4.5)	132 (5.8)	—
Smoking status, *n* (%)	—	—	—	—	—	—	<0.001
Current nonsmokers	15,287 (78.7)	1,402 (77.9)	3,114 (82.6)	8,490 (77.9)	1,014 (80.4)	1,267 (74.7)	—
Current smokers	4,247 (21.3)	404 (22.1)	690 (17.4)	2,483 (22.1)	245 (19.6)	425 (25.3)	—
Drinking status, *n* (%)	—	—	—	—	—	—	<0.001
Current nondrinkers	6,552 (27.8)	426 (20)	1,077 (23.4)	3,629 (28)	623 (46.2)	797 (41.4)	—
Current moderate drinkers	11,607 (64)	1,247 (71.3)	2,470 (68.8)	6,503 (63.2)	583 (48.7)	804 (53.1)	—
Current heavy drinkers	1,375 (8.2)	133 (8.7)	257 (7.8)	841 (8.9)	53 (5)	91 (5.5)	—
Physical activity, *n* (%)	—	—	—	—	—	—	<0.001
Yes	13,878 (75.5)	1,477 (84.3)	2,995 (81.8)	7,684 (73.7)	725 (61.9)	997 (64)	—
No	5,656 (24.5)	329 (15.7)	809 (18.2)	3,289 (26.3)	534 (38.1)	695 (36)	—
NHR, mean (SD)	3.14 (1.78)	2.20 (1.04)	2.55 (1.18)	3.44 (1.91)	3.66 (1.93)	3.78 (2.00)	<0.001
LHR, mean (SD)	1.57 (0.90)	1.18 (0.45)	1.33 (0.51)	1.73 (0.88)	1.66 (2.17)	1.64 (0.89)	<0.001
MHR, mean (SD)	0.42 (0.22)	0.31 (0.13)	0.36 (0.15)	0.46 (0.22)	0.50 (0.40)	0.51 (0.25)	<0.001

**Table 2:** The associations of three variables with four stages as a reference for stage 0 in weighted logistic regression models. Model was adujsted for age, gender, race, education, PIR, marital status, smoking status, drinking status, and physical activity.

Exposure	Stage 1	Stage 2	Stage 3	Stage 4	Advanced stages
	OR (95% CI)	OR (95% CI)	OR (95% CI)	OR (95% CI)	OR (95% CI)
NHR					
Continuous	1.95 (1.70, 2.22)	4.10 (3.64, 4.62)	9.13 (5.68, 14.7)	5.98 (4.37, 8.19)	1.85 (1.66, 2.06)
Q1	Reference	Reference	Reference	Reference	Reference
Q2	1.45 (1.17, 1.80)	2.62 (2.20, 3.12)	3.69 (1.83, 7.42)	2.95 (1.85, 4.71)	1.65 (1.34, 2.03)
Q3	2.48 (1.99, 3.09)	5.77 (4.76, 7.00)	12.5 (5.38, 29.20)	7.67 (4.85, 12.1)	2.09 (1.71, 2.55)
Q4	2.91 (2.31, 3.65)	20.30 (15.00, 27.30)	53.6 (17.9, 160)	36.10 (20.40, 63.70)	3.30 (2.69, 4.05)
*p* for trend	<0.001	<0.001	<0.001	<0.001	<0.001
LHR					
Continuous	2.14 (1.83, 2.50)	4.95 (4.39, 5.58)	4.82 (3.41, 6.81)	5.17 (3.80, 7.03)	1.29 (1.16, 1.43)
Q1	Reference	Reference	Reference	Reference	Reference
Q2	1.48 (1.20, 1.83)	2.16 (1.81, 2.56)	3.89 (2.19, 6.89)	2.35 (1.50, 3.69)	1.14 (0.96, 1.37)
Q3	2.02 (1.62, 2.52)	4.96 (4.01, 6.15)	4.87 (2.84, 8.34)	3.77 (2.38, 5.96)	1.27 (1.06, 1.51)
Q4	2.95 (2.35, 3.69)	26.10 (19.40, 35.2)	16.6 (8.57, 32.2)	25.70 (14.20, 46.60)	1.66 (1.36, 2.03)
*p* for trend	<0.001	<0.001	<0.001	<0.001	<0.001
MHR					
Continuous	1.92 (1.65, 2.24)	4.38 (3.89, 4.94)	8.1 (5.54, 11.8)	6.54 (4.76, 8.99)	1.84 (1.63, 2.08)
Q1	Reference	Reference	Reference	Reference	Reference
Q2	1.65 (1.35, 2.01)	2.23 (1.87, 2.67)	3.27 (1.57, 6.81)	2.16 (1.35, 3.45)	1.22 (1.02, 1.46)
Q3	2.12 (1.72, 2.63)	4.06 (3.34, 4.93)	7.98 (4.47, 14.2)	5.24 (3.37, 8.13)	1.88 (1.57, 2.24)
Q4	2.55 (1.98, 3.28)	16.20 (12.50, 21.10)	31.60 (14.40, 69.20)	29.60 (16.50, 53.10)	2.70 (2.21, 3.31)
*p* for trend	<0.001	<0.001	<0.001	<0.001	<0.001

## Data Availability

The Laboratory data from our study is publicly accessible online at https://wwwn.cdc.gov/nchs/nhanes/Default.aspx for global data users and researchers.

## Supporting Information

Supporting Information Table S1. Definitions of CKM conditions. Table S2. Methods for evaluating each CKM stage. Table S3. Detailed algorithm of the simplified 10-year CVD risk models. Figure S1. Subgroup analyses of the associations between NHR and advanced CKM stages. Models were adjusted for gender, age, race, education, PIR, marital status, drinking status, smoking status and physical activity.
