# Supplementary material for: Association of Three Composite Inflammatory and Lipid Metabolism Indicators With Cardiovascular-Kidney-Metabolic Syndrome: A Cross-Sectional Study Based on NHANES 1999–2020
Source: Mediators Inflamm. 2025 May 8;2025:6691516. doi: 10.1155/mi/6691516 (PMC12081155; doi:10.1155/mi/6691516)
Supplement: Supporting Information — Table S1. Definitions of CKM conditions. Table S2. Methods for evaluating each CKM stage. Table S3. Detailed algorithm of the simplified 10-year CVD risk models. Figure S1. Subgroup analyses of the associations between NHR and advanced CKM stages. Models were adjusted for gender, age, race, education, PIR, marital status, drinking status, smoking status and physical activity. [file 6691516.f1.docx]

**Supplementary Information**

**Title: Association of Three Composite Inflammatory and Lipid Metabolism Indicators with Cardiovascular-Kidney-Metabolic Syndrome: a cross-sectional study Based on NHANES 1999–2020**

**Author:** Jiayuan Song^1 #^, Ziyi Xu^2 #^, Han Yu^1^ , Aimin Li^1^, Yiying Liu^3^, Meiying Jin^1*^

**Supplementary Table 1 Definitions of CKM conditions**

| CKM conditions | Definition | CKM indicators | Threshold for CKM indicators |
| --- | --- | --- | --- |
| CVD | Individuals with clinical CVD or subclinical CVD | Clinical CVD | History of chronic heart failure, coronary heart disease, heart attack, or stroke |
|  |  | Subclinical CVD | Any of the following criterion is met:  1) Very high-risk CKD in KDIGO classification: UACR ≥ 300 mg/g and eGFR ≤ 45-59 ml/min/1.73m^2^, UACR ≥ 30 mg/g and eGFR ≤ 30-44 ml/min/1.73m^2^, or eGFR ≤ 29 ml/min/1.73m^2^.  2) Predicted 10-year CVD risk ≥ 20% |
| Kidney diseases | Individuals with CKD | CKD | Moderate-to-high-risk CKD in KDIGO classification: UACR ≥ 30 mg/g and eGFR ≥ 60 ml/min/1.73m^2^, UACR < 300 mg/g and eGFR ≤ 45-59ml/min/1.73m^2^, or UACR < 30 mg/g and eGFR ≤ 30-44 ml/min/1.73m^2^. |
| Metabolic disorders | Individuals with overweight/obesity,  abdominal obesity, prediabetes, diabetes, hypertension,  hypertriglyceridemia or MetS | Overweight/obesity | BMI ≥25 kg/m^2^ (or ≥23 kg/m^2^ if Asian ancestry) * |
|  |  | Abdominal obesity | Waist circumference ≥88/102 cm in female/male (or if Asian ancestry ≥80/90 cm in female/male) |
|  |  | Prediabetes | Fasting blood glucose ≥ 100-124 mg/dL or HbA1c ≥ 5.7%-6.4% and without self-reported diagnosis of diabetes, use of insulin, or oral hypoglycemic agents |
|  |  | Diabetes | Fasting blood glucose ≥ 125 mg/dL or HbA1c ≥ 6.5% or self-reported diagnosis of diabetes, use of insulin, or oral hypoglycemic agents |
|  |  | Hypertension | SBP ≥130 mm Hg or DBP ≥80 mm Hg or self-reported diagnosis of hypertension or use of antihypertensive medications |
|  |  | Hypertriglyceridemia | Triglycerides ≥ 135 mg/dL |
|  |  | MetS | MetS is defined by the presence of 3 or more of the following:  1) Waist circumference ≥88/102 cm in female/male (or if Asian  ancestry ≥80/90 cm in female/male).  2) HDL cholesterol ＜50/40 mg/dL in female/male.  3) Triglycerides ≥150 mg/dL.  4) Elevated blood pressure (SBP ≥130 mm Hg or DBP ≥80 mm Hg and/or use of antihypertensive medications)  5) Fasting blood glucose ≥100 mg/dL |

**Abbreviations:** BMI: body mass index; CKD: chronic kidney disease; CKM: cardiovascular-kidney-metabolic; CVD: cardiovascular disease; DBP: diastolic blood pressure; eGFR: estimated glomerular filtration rate; HDL: high-density lipoprotein; KDIGO: The Kidney Disease: Improving Global Outcomes; MetS: metabolic syndrome; SBP: systolic blood pressure; UACR: urinary albumin to creatinine ratio.

* Asian was not listed as a separate race/ethnicity until NAHNES 2011-2012, therefore the uniform threshold for BMI and waist circumference was used in all participants in NHANES 1999-2010.

**Supplementary Table 2 Methods for evaluating each CKM stage**

| CKM stages | Definition | Criterion | Threshold for CKM conditions |
| --- | --- | --- | --- |
| Stage 0: No CKM risk factors | Individuals with normal BMI and waist circumference,normoglycemia, normotension, a normal lipid profile, and no evidence of CKD or subclinical or clinical CVD | All criteria are met | BMI <25 kg/m^2^(or <23 kg/m^2^ if Asian ancestry) * |
|  |  |  | Waist circumference <88/102 cm in female/male (or if Asian ancestry <80/90 cm in female/male) |
|  |  |  | Fasting blood glucose < 100 mg/dL and HbA1c < 5.7% and without self-reported diagnosis of diabetes, use of insulin, or oral hypoglycemic agents |
|  |  |  | SBP <130 mm Hg and DBP <80 mm Hg without self-reported  diagnosis of hypertension or use of antihypertensive medications |
|  |  |  | HDL cholesterol ≥ 50/40 mg/dL in female/male and triglycerides < 150 mg/dL |
|  |  |  | Low-risk CKD in KDIGO classification according to eGFR and UACR: UACR < 30 mg/g and eGFR ≥ 60ml/min/1.73m^2^. |
|  |  |  | Predicted 10-year CVD risk < 20% |
|  |  |  | No clinical CVD |
| Stage 1: Excess or dysfunctional adiposity | Individuals with overweight/obesity, abdominal obesity, or dysfunctional adipose tissue, without the presence of other metabolic risk factors or CKD | Any of the three criteria is met | Overweight/obesity |
|  |  |  | Abdominal obesity |
|  |  |  | Prediabetes |
|  |  | All criteria are met | SBP <130 mm Hg and DBP <80 mm Hg without self-reported  diagnosis of hypertension or use of antihypertensive medications |
|  |  |  | HDL cholesterol ≥ 50/40 mg/dL in female/male and triglycerides < 150 mg/dL |
|  |  |  | Low-risk CKD in KDIGO classification according to eGFR and UACR: UACR < 30 mg/g and eGFR ≥ 60 ml/min/1.73m^2^. |
|  |  |  | Predicted 10-year CVD risk < 20% |
|  |  |  | No clinical CVD |
| Stage 2: Metabolic risk factors and CKD | Individuals with metabolic risk factors (hypertriglyceridemia, hypertension, MetS, diabetes), or CKD | Any of the five criteria is met | Hypertriglyceridemia |
|  |  |  | Hypertension |
|  |  |  | diabetes |
|  |  |  | MetS |
|  |  |  | Moderate-to-high-risk CKD in KDIGO classification |
|  |  | All criteria are met | No very high-risk CKD in KDIGO classification |
|  |  |  | Predicted 10-year CVD risk < 20% |
|  |  |  | No clinical CVD |
| Stage 3: Subclinical CVD in CKM | Subclinical CVD among individuals with excess/dysfunctional adiposity, other metabolic risk factors, or CKD | Any of the two criteria is met | Very high-risk CKD in KDIGO classification |
|  |  |  | Predicted 10-year CVD risk ≥ 20% |
|  |  | Any of the eight criteria is met | Overweight/obesity |
|  |  |  | Abdominal obesity |
|  |  |  | Prediabetes |
|  |  |  | Hypertriglyceridemia |
|  |  |  | Hypertension |
|  |  |  | diabetes |
|  |  |  | MetS |
|  |  |  | Moderate-to-high-risk CKD in KDIGO classification |
|  |  | The criterion is met | No clinical CVD |
| Stage 4: Clinical CVD in CKM | Clinical CVD among individuals with excess/dysfunctional adiposity, other metabolic risk factors, or CKD | The criterion is met | Clinical CVD |
|  |  | Any of the nine criteria is met | Overweight/obesity |
|  |  |  | Abdominal obesity |
|  |  |  | Prediabetes |
|  |  |  | Hypertriglyceridemia |
|  |  |  | Hypertension |
|  |  |  | diabetes |
|  |  |  | MetS |
|  |  |  | Moderate-to-high-risk CKD in KDIGO classification |
|  |  |  | Very high-risk CKD in KDIGO classification |

**Abbreviations:** BMI: body mass index; CKD: chronic kidney disease; CKM: cardiovascular-kidney-metabolic; CVD: cardiovascular disease; DBP: diastolic blood pressure; eGFR: estimated glomerular filtration rate; HDL: high-density lipoprotein; KDIGO: The Kidney Disease: Improving Global Outcomes; SBP: systolic blood pressure; UACR: urinary albumin to creatinine ratio.

* Asian was not listed as a separate race/ethnicity until NAHNES 2011-2012, therefore the uniform threshold for BMI and waist circumference was used in all participants in NHANES 1999-2010.

**Supplementary Table 3 Detailed algorithm of the simplified 10-year CVD risk models**

| **Women** | **log-Odds** = -3.307728 + 0.7939329 × (age – 55) /10 +  0.0305239 × (TC – HDL-C – 3.5) – 0.1606857 × (HDL-C –  1.3) /0.3 – 0.2394003 × (min(SBP, 110) – 110) /20 + 0.360078  × (max(SBP, 110) – 130) /20 + 0.8667604 × (if diabetes) +  0.5360739 × (if current smoker) + 0.6045917 × (min(eGFR,  60) – 60) / -15 + 0.0433769 × (max(eGFR, 60) – 90) / -15 +  0.3151672 × (if using anti-hypertensive medication) –  0.1477655 × (if using statin) – 0.0663612 × (if using antihypertensive medication) × (max(SBP, 110) – 130) /20 +  0.1197879 × (if using statin) × (TC – HDL-C – 3.5) –  0.0819715 × (age – 55) /10 × (TC – HDL-C – 3.5) +  0.0306769 × (age – 55) /10 × (HDL-C – 1.3) /0.3 – 0.0946348  × (age – 55) /10 × (max(SBP, 110) – 130) /20 – 0.27057 ×  (age – 55) /10 × (if diabetes) – 0.078715 × (age – 55) /10 × (if  current smoker) – 0.1637806 × (age – 55) /10 × (min(eGFR,  60) – 60) / -15  **Risk** = exp(log-Odds) / (1 + exp(log-Odds)) |
| --- | --- |
| **Men** | **log-Odds** = -3.031168 + 0.7688528 × (age – 55) /10 +  0.0736174 × (TC – HDL-C – 3.5) – 0.0954431 × (HDL-C –  1.3) /0.3 – 0.4347345 × (min(SBP, 110) – 110) /20 +  0.3362658 × (max(SBP, 110) – 130) /20 + 0.7692857 × (if  diabetes) + 0.4386871 × (if current smoker) + 0.5378979 ×  (min(eGFR, 60) – 60) / -15 + 0.0164827 × (max(eGFR, 60) –  90) / -15 + 0.288879 × (if using anti-hypertensive medication)  – 0.1337349 × (if using statin) – 0.0475924 × (if using antihypertensive medication) × (max(SBP, 110) – 130) /20 +  0.150273 × (if using statin) × (TC – HDL-C – 3.5) – 0.0517874  × (age – 55) /10 × (TC – HDL-C – 3.5) + 0.0191169 × (age –  55) /10 × (HDL-C – 1.3) /0.3 – 0.1049477 × (age – 55) /10 ×  (max(SBP, 110) – 130) /20 – 0.2251948 × (age – 55) /10 × (if  diabetes) – 0.0895067 × (age – 55) /10 × (if current smoker) –  0.1543702 × (age – 55) /10 × (min(eGFR, 60) – 60) / -15  **Risk** = exp(log-Odds) / (1 + exp(log-Odds)) |

**Abbreviations:** eGFR: estimated glomerular filtration rate; HDL: high-density lipoprotein cholesterol; SBP: systolic blood pressure; TC: total cholesterol.


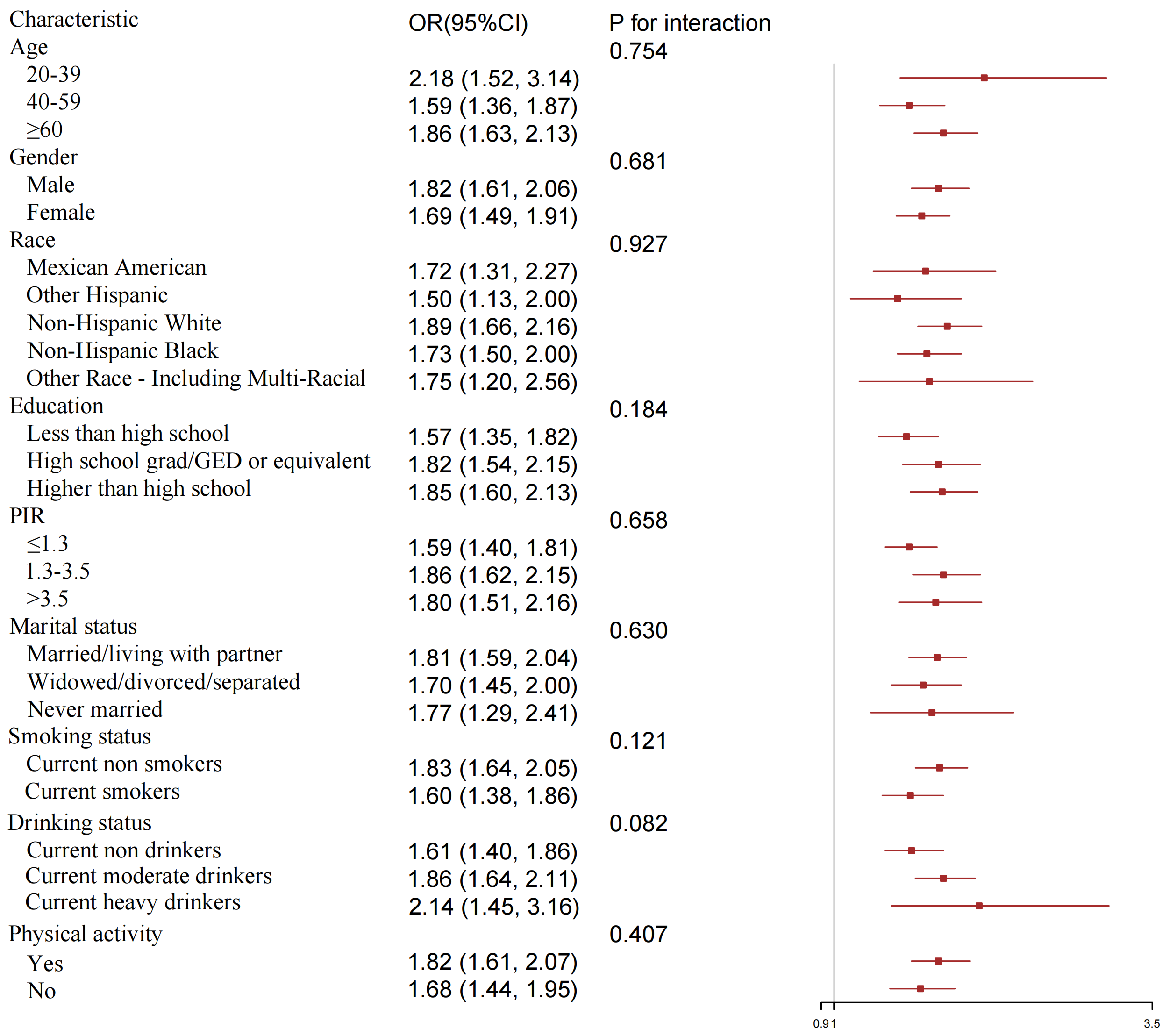


**Supplementary Figure 1** Subgroup analyses of the associations between NHR and advanced CKM stages. Models were adjusted for gender, age, race, education, PIR, marital status, drinking status, smoking status and physical activity.
